# Sensitivity, Specificity, and Accuracy of Color Doppler Ultrasonography for Diagnosis of Retinal Detachment

**DOI:** 10.18502/jovr.v15i2.6733

**Published:** 2020-04-06

**Authors:** Mohammadreza Akhlaghi, Masoomeh Zarei, Majid Ziaei, Mohsen Pourazizi

**Affiliations:** ^1^ Department of Ophthalmology, Isfahan Eye Research Center, Isfahan University of Medical Sciences, Isfahan, Iran; ^2^ Nafis Private Imaging Center, Isfahan, Iran; ^3^ Pediatric Inherited Diseases Research Center, Research Institute for Primordial Prevention of Non-Communicable Disease, Isfahan University of Medical Sciences, Isfahan, Iran

**Keywords:** Color Doppler Ultrasonography, Retina, Retinal Detachment

## Abstract

**Purpose:**

This study evaluated the sensitivity, specificity, and diagnostic accuracy of Color Doppler Ultrasonography (CDUS) in patients with suspected retinal detachment (RD) who underwent surgery.

**Methods:**

In this prospective, observational clinical study, 65 eyes of 65 consecutive patients with suspected RD with opaque media were included. Following a standardized protocol, CDUS of the retina of the affected eye was performed. The sensitivity, specificity, and diagnostic accuracy of CDUS were determined and compared to the findings during surgery.

**Results:**

The mean age of patients (18 men and 47 women) was 52.36 years (range: 8–77 years). The sensitivity, specificity, and overall accuracy of ocular CDUS were 91.3%, 88.1%, and 89.2%, respectively. The false-negative rate (negative CDUS images but presence of RD at operation) was 3.1% (2/65) and the false-positive rate (positive CDUS images but absence of RD at operation) was 7.7% (5/65).

**Conclusion:**

CDUS of the retina could be considered as a promising tool in the diagnosis of RD in patients with opaque media.

##  INTRODUCTION

Retinal detachment (RD) is a serious eye disease with the potential risk of blindness. It requires immediate management to prevent permanent vision loss.^[1,2]^ Accurate diagnosis of RD may be difficult in some cases and is important as it affects the prognosis and treatment plan.^[3]^

It is recommended that ophthalmologists rely on their clinical skills, including indirect ophthalmoscopy and biomicroscopic examination of the fundus with slit lamp, for the diagnosis of RD in suspected cases.^[1]^

In the presence of dense media opacities (e.g., corneal edema, hyphema, cataract, vitreous hemorrhages) with clinical suspicion of RD, the diagnosis may not be accurate, and poor visual prognosis can be expected if the ophthalmologists wait for the media to clear.^[4,5]^ Moreover, the decision to perform surgery in all clinically suspected cases poses additional costs on patients and the healthcare system. Therefore, alternative diagnostic modalities, such as ocular ultrasound, are required in this clinical situation.^[6]^ Gray-scale ultrasound can identify RD in suspected cases with dense media opacities.^[7]^

Although gray-scale ultrasound can differentiate RD from other membranous structures in the presence of opaque media, it has some limitations that may lead to a diagnostic dilemma.^[6,8]^ Differentiating individual retinal layers is not possible on gray-scale ultrasound so it may be difficult to accurately differentiate retinoschisis from RD.^[9]^ The posterior hyaloid membrane or central vitreous gel are not usually attached to the optic disc and are visualized as structures with weaker echogenicity and more variable thickness, with a greater mobility than RD. However, differentiating RD from those structures in the posterior segment is difficult in cases of shallow or localized RD.^[10,11]^

In such cases, color Doppler ultrasound (CDUS) can play an important role in the diagnosis of RD by demonstrating blood flow in the detached retina. CDUS helps ophthalmologists examine the retinal blood flow, even in the presence of dense ocular opacities preventing a direct view of the posterior segment.^[12]^ The detached retina is seen on CDUS as a curvilinear structure in the vitreous cavity with blood flow.^[8,12]^ Therefore, CDUS can play an additional and more reliable role in the diagnosis through visualization of flow signals in the detached retina.^[8]^

However, despite CDUS having several potential benefits, a limitation of the application of CDUS in the diagnosis of RD is that the evidence of its diagnostic accuracy is scattered and limited. The aim of this study was to evaluate the clinical utility and diagnostic value of CDUS in the detection of RD in patients with dense ocular opacities.

**Figure 1:**
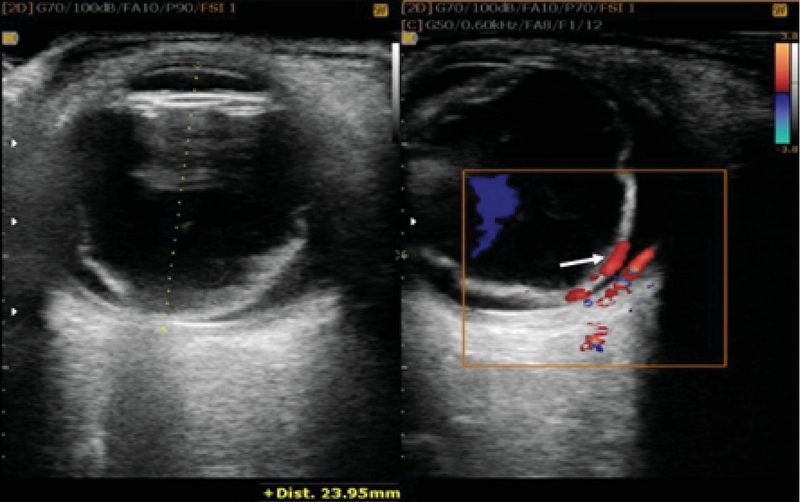
(Left) The funnel-shaped membrane has been marked on the gray-scale B scan. (Right) The figure shows arterial flow and retinal detachment.

**Figure 2:**
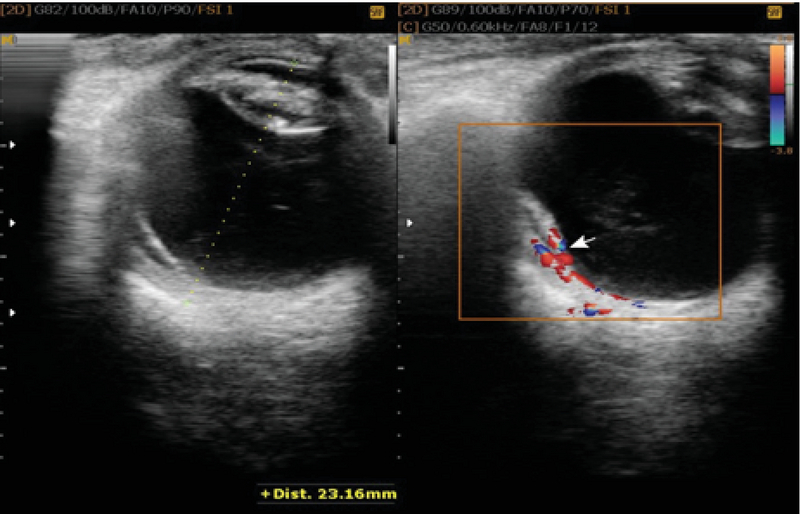
(Left) The gray-scale image of the horizontally scanned eye demonstrates central partial retinal detachment. (Right) Color Doppler ultrasound reveals thick linear and spotty color signals.

**Figure 3:**
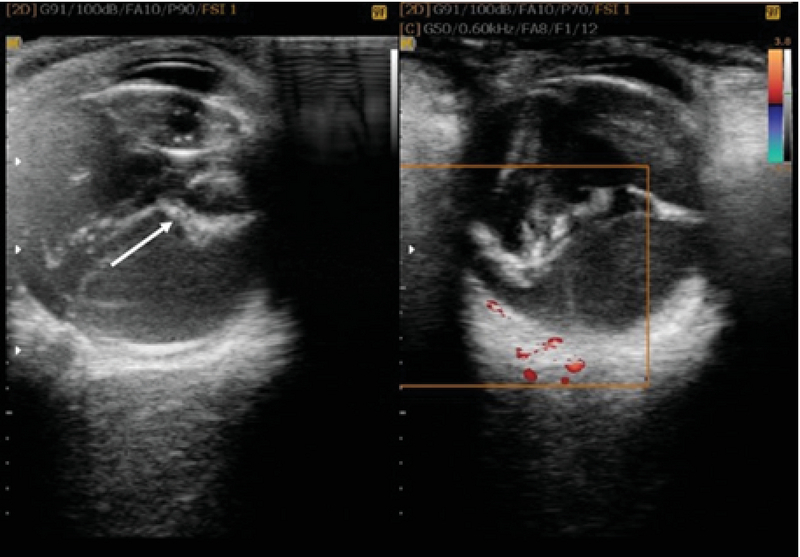
Color Doppler ultrasonography shows no flow in the membrane seen in the left figure, ruling-out RD. The avascular membrane has been marked in the right figure.

##  METHODS

### Patients and Study Design 

This prospective, observational clinical study was approved by the Ethics Committee of Isfahan University of Medical Sciences. The study protocol adhered to the tenets of the Declaration of Helsinki. Signed informed consent was obtained from participants prior to the study. We enrolled 65 eyes of 65 patients with opaque media who were candidates for ocular surgery to clear the media and repair RD if present (pars plana vitrectomy +/– phacoemulsification, with or without corneal graft, etc.). Patients who were inoperable because of any medical, surgical, or general condition and those with a past or current history of tumors in the vitreous cavity were excluded.

### Doppler Ultrasound Imaging 

On the day of enrollment, CDUS of the eye was performed for all individuals by an expert radiologist trained in ultrasound of the retina using a CDUS unit and 13-MHz linear array transducer, Medison V20 (Medison Co. Ltd., Seoul, Korea). The evaluation techniques have been described previously.^[8]^ Patients were examined in the supine position to avoid any pressure on the eye. Sterile coupling gel was applied to the closed eyelids, with the examiner's hand resting on the orbital margin to minimize pressure on the globe, and color flow images were obtained.^[8]^

RD was described as visualization of a flow signal (presence of vascularity) along the detached retina, while vitreous membranes were described as lack of vascularity.^[10]^

### Reference Standard

Fundus examination during surgery was considered as the gold standard for the diagnosis of RD. The final clinical diagnosis of RD was made by two expert ophthalmologists (first and second authors) during surgery.

### Statistical Analysis

Statistical analyses were performed by a statistician using the SPSS software, version 16.0 (SPSS, Chicago, IL, USA). Sensitivity and specificity were calculated for diagnostic CDUS, with the final clinical diagnosis during the operation as the reference standard. Sensitivity was calculated as the proportion of patients with actual RD who had an abnormal retina on CDUS. Specificity was calculated as the proportion of patients with no actual RD who had a normal retina on CDUS. Accuracy was calculated as the proportion of patients whose RD status was correctly predicted using ultrasound.

##  RESULTS

In this study, we performed CDUS for 65 eyes of 65 patients, including 18 men and 47 women, suspected with RD. The mean age of patients was 52.36 years (range: 8–77 years). Vitreous hemorrhage, cataract, total hyphema, and corneal opacity were detected in 44 (67.7%), 12 (18.58%), 6 (9.2%), and 3 eyes, respectively.

Of the 65 patients, RD was diagnosed in 26 (40%) patients on CDUS and in 23 (35.4%) patients during vitrectomy. As for the type of RD, all patients had rhegmatogenous RD.

The sensitivity, specificity, and overall accuracy of ocular CDUS were 91.3%, 88.1%, and 89.2%, respectively. The false-negative rate (negative with CDUS but positive with operation) was 3.1% (2/65) and the false-positive rate (positive with CDUS but negative with operation) was 7.7% (5/65). False-positive cases included four cases of severe neovascular membrane in proliferative diabetic retinopathy and one case of posterior vitreous detachment.

##  DISCUSSION

The current study demonstrated that CDUS is sensitive and specific for the diagnosis of RD in patients with dense ocular opacities. In most cases, gray-scale B-mode ultrasonography allows differentiation of a total RD from a vitreous membrane; however, this differentiation may be challenging in some situations.^[4,13,14]^ Cases of partial RD and vitreous membrane can share similar ultrasonographic features.^[4,5][14]^ In patients with atypical findings on gray-scale ultrasound of shallow RD, CDUS can play an additional and more reliable role in the diagnosis by enabling detection of blood flow in the detached retina.^[13,15]^

Advantages of performing CDUS to diagnose RD are that CDUS can be a quick, noninvasive, and safe method for detecting total and partial RDs. CDUS enables ophthalmologists to examine ocular blood flow, even in the presence of dense ocular opacities preventing a direct view to the posterior segment of the eye.^[12]^

A previous study at Isfahan Eye Research Center by Ghanbari et al indicated the diagnostic data of gray-scale sonography as follows: sensitivity, 87.5%; specificity, 64.5%; and accuracy, 72.4%.^[16]^

In the current study performed at the same center, the sensitivity, specificity, and overall accuracy of ocular CDUS compared to surgical findings were 91.3%, 88.1%, and 89.2%, respectively. These differences can be explained by limitations of gray-scale ultrasound in detection of RD as previously mentioned. There is no quick, noninvasive, and safe gold standard for the diagnosis of RD. Although B-scan has the aforementioned characteristics, there are some limitations.^[8,16]^

Studies examining changes in ocular blood flow velocities in RD are limited.^[10]^ Ido et al found the usefulness of CDUS in the diagnosis of RD in the presence of hazy media.^[10]^ Similar to ours, in their study, the absence or presence of RD was confirmed during surgery. Their study on 33 consecutive patients demonstrated a sensitivity of 92.3%, a specificity of 100%, a positive predictive value of 100%, a negative predictive value of 93.3%, and an accuracy of 96.3%. In their study, all patients with blood flow on CDUS were confirmed to have RD during surgery.^[10]^

In the study by Han et al, CDUS showed a color signal in approximately 60% of RD cases.^[8]^ Wong et al reported the sensitivity of CDUS to be 100%.^[17]^ The sensitivity and specificity of ocular CDUS were both approximately 90% in our study. The difference between these results can be explained by certain factors, including the type of RD, duration of detachment, coexisting pathologies, etc.

The ability of CDUS to demonstrate flow in vascular structures and subsequently yield the diagnosis of RD in patients with dense ocular opacities depends on factors including flow velocity, vessel size, depth of the lesion, scanner sensitivity, and operator control.^[8]^ Therefore, the sensitivity, specificity, and accuracy can be affected.^[8]^ Most longstanding RDs are peripheral, not involving the posterior pole. A reason for the difference in results of similar studies in this field is the presence of longstanding RD. CDUS has a lower ability to detect blood flow in the retinal periphery. This may be a possible explanation for the false-negatives of CDUS.^[8]^

Despite several potential benefits, the interobserver variability could be a possible explanation for CDUS not being routinely used in ophthalmic practice.^[18]^ To increase the detectability of Doppler signals, several kinds of ultrasound contrast agents can be used. In the study by Han et al, the sensitivity of CDUS to detect flow in RD increased from approximately 60% to 90% after intravenous contrast administration.^[8]^ Although a highly accurate diagnosis is achieved using contrast-enhancing agents, the procedure would be invasive.

In our study, two patients who were negative for RD on CDUS were diagnosed with RD based on surgical data. We did not find any meaningful difference between the characteristics of these patients and others, for example, regarding the type of RD. Negative results on CDUS but positive results during operation may occur in the presence of any ischemic event in the retinal vessels, including arterial and venous occlusions.

Of the false-positive cases in the current study, four cases were of severe neovascular membrane in proliferative diabetic retinopathy and one case was of posterior vitreous detachment. There were no cases of traction RD.

The overall accuracy of CDUS was approximately 90%. In the study by Wong et al,^[17]^ all patients with RD were diagnosed using CDUS, consistent with our results.

There are some limitations in the current study, including the relatively small sample size. Relative afferent pupillary defects were not recorded in this study, and their relationship with other findings was not evaluated. Failure to use contrast-enhanced CDUS is another shortcoming of the current study. The lower ability of CDUS to detect blood flow in the retinal periphery is an inherent limitation of the technique. The prospective nature of this study on the accuracy of CDUS in diagnosing RD in eyes with dense ocular opacities and suspected RD is a strength of the current study.

In conclusion, CDUS helps in distinguishing between RD and vitreous membrane in eyes with opaque media when the results of B-scan sonography are inconclusive. Large, prospective studies are required to confirm the greater accuracy of CDUS compared to other modalities in the diagnosis of RD in these patients.

##  Financial Support and Sponsorship

None.

##  Conflicts of Interest

There are no conflicts of interest.
